# A Single Experiment

**Published:** 1868-11

**Authors:** 


### Editorial.

### "A SINGLE EXPERIMENT."

"Do you communicate with Watt? Tell him I have found by a single experiment, that nitrous oxide, nor any per centage of it, in inhalation, *enters the blood at all.*" Neither its oxygen nor its other component. What will he say to *this?* Of course, devotee of truth as he is, errors of long standing to the contrary notwithstanding, won't deny this.

"I should like to hear from him upon the subject. At least what he says thereupon."

The above is stolen from a private letter. It is the language of a distinguished physiologist. Now if he has "found" that, it is all right. Just so it is "*found*" it matters not whether by "a single experiment" or by a dozen. It is hoped that by "another of the same" he will find what sustains the respiration while breathing the oxyd (atmospheric air being rigidly excluded) for five, ten and *twenty* minutes, or for an hour with less than a dozen breaths of air. W.

